# Supplementary material for: Parenteral Nutrition in Patients with Incurable Cancer: Exploring the Heterogenous and Non-Randomised Clinical Landscape
Source: Curr Oncol. 2025 Nov 18;32(11):644. doi: 10.3390/curroncol32110644 (PMC12651192; doi:10.3390/curroncol32110644)
Supplement: Supplementary file 1 [file curroncol-32-00644-s001.zip › PATNIC_supplementary_17.11.2025.pdf]

Parenteral Nutrition in Patients with Incurable Cancer: Exploring the Heterogenous and Non-randomised Clinical Landscape
# - Supplementary material

Section 1. Supplementary information on patient characteristics

Cancer disease

This table presents a list of location of metastasis for those 84% of patients (*n*=427) who had metastasis at parenteral nutrition (PN) start.

Supplementary table S1. If metastases, location of metastasis

Location of metastasis at PN start[1]	*n*=427, *n* (%)
Liver	168 (39%)
Lymph nodes	157 (37%)
Peritoneal carcinomatosis	150 (34%)
Peritoneum mesentery	101 (24%)
Lung	77 (18%)
Bone	63 (14%)
Central nerve system	15 (3%)
Other	108 (21%)
Upper gastrointestinal tract[2]	17 (4%)
Adrenal gland	16 (4%)
Colorectal	15 (4%)
Spleen	14 (3%)
Pelvis	13 (3%)
Skin	6 (1%)
Ovaries/uterus	6 (1%)
Heart	5 (1%)
Abdominal area	4 (1%)
Pleura	4 (1%)
Prostate	2 (<1%)
Breast	1 (<1%)
Kidney	1 (<1%)
Soft tissue	1 (<1%)
Not specified[3]	12 (3 %)

PN=parenteral nutrition. *n* indicates number of cases. [1]Option that patients had several metastases. .
[2]Esophageal cancer, stomach cancer, pancreatic cancer, liver cancer, gallbladder cancer [3] Information not found.

This table lists new metastases for those 20% of patients (*n*=101) where new metastasis was identified during the time the patients received parenteral nutrition treatment.

Supplementary table S2. New metastasis during parenteral nutrition treatment

Location of new metastasis[1]	*n*=101, *n* (%)
Liver	30 (30%)
Lymph nodes	10 (10%)
Peritoneal carcinomatosis	30 (30%)
Peritoneum mesentery	17 (17%)
Lung	14 (14%)
Bone	12 (12%)
Central nerve system	7 (7%)
Other	21 (21%)

[1]Option that patients had several new metastases.

Cancer treatment during the time patients received parenteral nutrition

During the period when patients received parenteral nutrition (PN), patients both started and discontinued anti-cancer therapy. This table lists the initiation of new therapy and the number of patients discontinuing therapy during PN (*n*= 238, 47%).

Supplementary table S3. Cancer therapy during PN treatment

Cancer treatment during PN treatment	*n = 238, n* (%)
Started anticancer therapy[1]	107 (21%)
Chemotherapy	70 (62%)
Radiotherapy	23 (20%)
Surgery	11 (9%)
Immunotherapy	4 (4%)
Targeted therapy	9 (8%)
Other	5 (5%)
Discontinued anticancer therapy	164 (32%)

PN= Parenteral nutrition. *n* indicates number of cases. [1]Combination of several therapies possible.

Biochemical blood analysis

Supplementary table S4. Biochemical blood analysis at start of parenteral nutrition

Parameter	*n (%)*	Mean (SD)	Median (IQR)	Normal range (*m/w*)
Sodium, mmol/L	494 (97%)		137 (134-140)	137-145
Potassium, mmol/L	494 (97%)	3.87 (0.56)		3.6-5
Calcium, mmol/L	371 (73%)	2.24 (0.22)		2.2-2.55
Creatinine, µmol/L	477 94%)		64 (49-83)	60-105/45-90
Phosphate, mmol/L	136 (27%)	1.02 (0.28)		0.75-1.65/0.85-1.5
Magnesium, mmol/L	222 (44%)	0.79 (0.17)		0.71-0.94
Haemoglobin, g/dL	491 (97%)	11.44 (1.68)		13.4-17/11.7-15.3
Leukocytes, 10^9/L	490 (97%)		8.6 (5.9-11.5)	3.5-10
Neutrophils, 10^9/L	359 (71%)		6.2 (3.6-9.75)	1.5-7.3
Lymphocytes, 10^9/L	211 (42%)		1.1 (0.7-1.65)	1.1-3.3
Thrombocytes, 10^9/L	451 (89%)		277 (191-398.5)	145-348/265-387
Glucose, mmol/L	337 (66%)		6.1 (5.3-7.3)	4-6
CRP, mg/L	487 (96%)		52.0 (17–107)	< 5
Albumin, g/L	438 86%)	31.86 (6.84)		34-48
Urea, mmol/L	132 (26%)		5.8 (4.3-7.9)	3.2-8.1

m/w indicates men and women. *n* indicates number of cases.

Comorbidities

Comorbidities were recorded at the initiation of parenteral nutrition (PN) treatment. The Charlson Comorbidity Index was used to classify comorbidities. Other comorbidities were reported as free text. No comorbidities were reported for 36% of patients, and for six patients (<1%), information was not found. For the remaining 63% of patients, one to six comorbidities were identified. The two tables below provide lists of the types and numbers of comorbidities at the initiation of PN treatment (*n*=507).

Supplementary table S5. Comorbidities at start of parenteral nutrition

Comorbidities	*n* = *507, n* (%)
Myocardial infarct/ coronary disease	24 (5%)
Heart failure	19 (4%)
Other cardiac diseases	
Arrhythmias	19 (4%)
Heart transplant	1 (<1%)
Ventricular septal defect	1 (<1%)
Heart valve stenosis/insufficiency	6 (1%)
Peripheral vascular disease	27 (5%)
Cerebrovascular disease	7 (1%)
Hypertension	68 (13%)
Connective tissue disease	53 (10%)
Chronic pulmonary disease	53 (10%)
Diabetes	38 (7%)
Moderate or severe renal disease	31 (6%)
Mild liver disease	4 (1%)
Moderate or severe liver disease	9 (2%)
Neurological disease (e.g. multiple sclerosis, epilepsy)	10 (2%)
Psychiatric condition (e.g. anxiety, depression)	14 (3%)
Thyroidal disorders (e.g. hyper- or hypothyroidism)	23 (5%)
Gastrointestinal conditions	
Ulcer disease	12 (2%)
Reflux/gastritis	11 (%2)
Inflammatory bowel syndrome	6 (1%)
Diverticulitis	4 (1%)
Ileus and sub-ileus	3 (<1%)
Short bowel syndrome	2 (<1%)
Celiac disease	2 (<1%)
Chronic pancreatitis	2 (<1%)
Enterocutaneous fistula	1 (<1%)
Cholangitis	1 (<1%)
Gastrectomy	1 (<1%)
Osteoporosis	8 (2%)
Second cancer[1]	5 (1%)
Hemiplegia	1 (<1%)
Dementia/ cognitive disability	3 (<1%)
Psoriasis	4 (1%)
Anaemia	3 (<1%)
Gout	2 (<1%)
GVHD	2 (<1%)
Dyslipidaemia	1 (<1%)
Aorta dissection	1 (<1%)
Myalgic encephalomyelitis	1 (<1%)
Not specified[2]	6 (1%)
None	182 (36%)

n indicates number of cases. [1] Leukaemia 3, Metastatic prostate cancer 2, [2]Information not found.

Supplementary table S6. Number of comorbidities

Number of comorbidities	*n = 507, n* (%)
0	182 (36%)
1-2	283 (56%)
3-4	31 (6%)
5-6	5 (1%)
Not specified[1]	6 (1%)

n indicates number of cases.[1]Information not found.

Medications

Medications were recorded at the initiation of parenteral nutrition (PN) treatment. For three patients, information was not found (<1%). The two tables below provide a list of the available information about types and numbers of medications at the initiation of PN treatment (*n*=507).

Supplementary table S7. Medications at start of parenteral nutrition

Medications	*n* = 507, *n* (%)
Non-opioid analgesics	335 (66%)
Opioids	318 (63%)
Co-analgesics, not specified	32 (6%)
Corticosteroids	158 (31%)
Anticholinergics	362 (71%)
Antidepressants	59 (12%)
Neuroleptics	66 (13%)
Hypnotics/anxiolytics	225 (44%)
Antiepileptic	19 (4%)
Antiemetics	287 (57%)
Pancreatic enzymes	26 (5%)
Drug(s) for acid related disorders[1]	314 (62%)
Laxatives	246 (49%)
Anti diarrheal	36 (7%)
Antibiotics	157 (31%)
Antimycotics	54 (11%)
Antivirals	7 (1%)
Antihypertensives	164 (32%)
Anticoagulant/antithrombotic	95 (19%)
Diuretics	95 (19%)
Other heart medications	41 (8%)
Antihistamine	24 (5%)
Uric acid inhibitor	12 (2%)
Bisphosphonates	11 (2%)
Colony stimulating factor	5 (1%)
Spasmolytics	4 (1%)
Not specified[2]	3 (<1%)
Antiflatulent agent	4 (1%)
None	3 (<1%)
Other	9 (2%)

n indicates number of cases.[1]Alginate, Antacids, H2 –receptor antagonists (Cimetidin, Famotidine, Ranitidin), PPIs4 (Esomeprazol, Lansoprazol, Pantoprazol), Nexium, Somac Control. [2]Information not found.

Supplementary table S8. Number of medications

Number of medications	*n* = 507, *n* (%)
0	3 (<1%)
1-3	97 (19%)
4-6	193 (38%)
7-9	161 (32%)
10-15	50 (10%)
Not specified	3 (<1%)

n indicates number of cases. [1]Information not found.

Ascites

The presence of ascites was documented both at the initiation and during parenteral nutrition (PN) treatment (*n*=229, 45%). The status was graded throughout the PN treatment period and is presented in the table below.

Supplementary table S9. Ascites at start and during parenteral nutrition treatment

Variable	*n* =229, *n* (%)
Presence of ascites at PN start (yes)	155 (30%)
Presence of ascites during PN (yes)	203 (40%)
Status of ascites during PN	
Newly formed/ formed after PN start	67 (33%)
Worsened from PN start	54 (26%)
Same status as PN start	45 (22%)
Status not described	34 (17%)
Other	5 (2%)

PN=parenteral nutrition. *n* indicates number of cases.

SECTION 2. SUPPLEMENTAL INFORMATION OF THE PARENTERAL NUTRITION TREATMENT

Parenteral nutrition treatment

This table presents data on the administration of parenteral nutrition (PN), including the route of infusion, infusion schedule, and PN solution.

Supplementary table S10. Parenteral nutrition infusion

Variable	*n*=507, *n* (%)
Route of infusion	
Central infusion	
Hickman tunnelled catheter	121 (24%)
Central venous catheter	126 (25%)
Short term central venous catheter (CVK)	65 (13%)
Transthoracic venous port	187 (37%)
Peripheral infusion	
Peripherally inserted central catheter	19 (4%)
Peripheral venous catheter	181 (38%)
Infusion schedule of PN	
Continuous infusion	26 (5%)
Nightly cyclical infusion	179 (35%)
Cyclical infusion, other	274 (54%)
Not specified[1]	28 (6%)
PN solution[2]	
SmofKabiven	429 (85%)
Kabiven	5 (1%)
Nutriflex	22 (4%)
OliClinomel	14 (3%)
Smofkabiven, electrolyte free	1 (<1%)
Smofkabiven, fat free	1 (<1%)

PN=parenteral nutrition. *n* indicates number of cases. Data are presented in median (min-max)., [1] Information not found. [2]Data available for *n*=472.

Drainage of gastric fluid

Drainage of gastric fluid was documented at the start of parenteral nutrition treatment and is presented in the following table.

Supplementary table S11. Drainage of gastric fluid at PN start

Drainage of gastric fluid at PN start	*n*=507, *n* (%)
No drainage of gastric fluid	327 (65%)
Nasogastric tube	120 (24%)
Percutaneous endoscopic gastrostomy (PEG)	23 (4%)
Not specified[1]	37 (7%)

PN=parenteral nutrition. *n* indicates number of cases. [1]Information not found.

Median and max doses of parenteral nutrition

The table below presents the difference between the median and maximum doses of parenteral nutrition prescribed and the doses actually received by the patient. For 2% of patients, no information was found.

Supplementary table S12. Median and max doses of parenteral nutrition treatment

**Variable**[1]	***n*=507, *n* (%)**	**Median (range)**
Dose ordinated (kcal/day)	404 (80%)	1100 (275–2200)
Dose given (kcal/day)	497 (98%)	1050 (0-2200)
Difference in dose ordinated and given (kcal/day)	404 (80%)	125 (-550-1100)
Max dose ordinated (kcal/day)	404 (80%)	1600 (490-2700)
Max dose given(kcal/day)	497 (98%)	1600 (1100-2900)
Difference in max dose ordinated and given	404 (80%)	0 (-900–1200)
Number of adjustments in dose	409 (80%)	3 (1-15)

n indicates number of cases. kcal=kilocalories. [1]Data might be missing or information not found.

Additional vitamins, minerals and other additives

The table below presents the additives administered alongside parenteral nutrition.

Supplementary table S13. Additives given with parenteral nutrition

**Supplement** [1]	***n*=507, *n* (%)**
Initiative vitamin supplements	
Pabrinex®	70 (114%)
Thiamine	29 (6%)
Added vitamins and minerals to PN bags[2]	
Soluvit®	481 (95%)
Vitalipid Adult®	479 (94%)
Cernevit®	1 (<1%)
Addaven®	476 (94%)
Electrolyte correction	
Potassium chloride	76 (15%)
Monopotassium phosphate	21 (4%)
Magnesium sulphate	6 (1%)
Calcium chloride	5 (1%)
Other additives	
Insulin	14 (3%)
Dipeptiven® (alanine + glutamine)	11 (2%)
Premixel®	4 (<1%)
Albumin	1 (<1%)

PN=parenteral nutrition. *n* indicates number of cases. [1]Dose not specified. [2]Standard supplements might be given to all but data was missing in the medical records.

Additional oral supplements

The table below presents the oral supplements administered alongside parenteral nutrition.

Supplementary table S14. Oral supplements given with parenteral nutrition

**Supplement** [1]	***n*=507, *n* (%)**
Magnesium	44 (9%)
Potassium	52 (10%)
Calcium	26 (5%)
Berocca®[2]	7 (1%)
**Other**	
Multivitamin	45 (9%)
Vitamin B-complex[3]	11 (%)
Vitamin B6	3 (<1%)
Folic acid	11 (2%)
Calcigran Forte®[4]	8 (2%)
Vitamin D	22 (4%)
Vitamin B12	18 (5%)
Vitamin C	2 (<1%)
Vitamin E	1 (<1%)
Phosphate	35 (7%)
Iron	5 (1%)
Kajos®[5]	7 (1%)
Potassium Chloride	9 (2%)
Calcium pantothenate	1 (<1%)
NaCl tablet	4 (<1%)
Sodium	1 (<1%)
N-3 PUFAs[6]	4 (<1%)
Cod liver oil (including A, D, E)	2 (<1%)
Lipofundin®	1 (<1%)
Beta-Sitosterol®	1 (<1%)
GEM - glucose®	1 (<1%)

n indicates number of cases. [1]Dose not specified. [2]Berocca contains Vitamin C, B1, B2, B3, B5, B6, B12, Biotin, Folic Acid, Calcium, Magnesium, Zink, Sodium. [3]Nycoplus Vitamin B (B1, B2, B3, B5, B6, biotin, folic acid, B12) n=3, TrioBe (folic acid, B12, B6) n=5, B-tonin (B1, B2, B3, B5, B6, caffeine) n=2, Afi-B total (B1, B2, B3, B5, B6) n=1, B2, B3, B6 n=1. [4]Calcigren forte contains calcium and vitamin D. [5]Kajos contains potassium.[6]Omega 3 supplement.

Additional intravenous (IV) treatment

The table below presents additional intravenous (IV) fluids administered during parenteral nutrition treatment. The table includes only the starting dose and does not specify the duration of treatment or any adjustments in doses.

Supplementary table S15. Additional intravenous (IV) treatment

**Registered additional fluid (starting dose)**	***n=507, n* (%)**	**Median (IQR)**
Additional IV-fluid	475 (93%)	
Ringer Acetate (ml)	305 (60%)	1000 (1000–1000)
Sodium chloride (ml)	385 (76%)	1000 (1000–1000)
Glucose (ml)	113 (22%)	1000 (500–1000)
Carbolyte (ml)	3 (<1%)	1000 (500-1000)

n indicates number of cases.

Previous PN treatment

The table below presents the number of patients who have previously received parenteral nutrition (PN) treatments, as well as the frequency of PN administration for each patient.

Supplementary table S16. Previous parenteral nutrition treatment

**Variable**	***n=507, n* (%)**
Previous PN treatments (yes)	292 (58%)
Unknown	30 (6%)
Number of times	
1	89 (31%)
2	66 (23%)
3	19 (6%)
4	4 (1%)
Unknown	114 (39%)

PN=parenteral nutrition. *n* indicates number of cases.
